# Intensivists’ beliefs about rapid multiplex molecular diagnostic testing and its potential role in improving prescribing decisions and antimicrobial stewardship: a qualitative study

**DOI:** 10.1186/s13756-021-00961-4

**Published:** 2021-06-29

**Authors:** Alyssa M. Pandolfo, Robert Horne, Yogini Jani, Tom W. Reader, Natalie Bidad, David Brealey, Virve I. Enne, David M. Livermore, Vanya Gant, Stephen J. Brett, the INHALE WP2 Study Group, Julie Barber, Laura Shallcross, Jeronimo Cuesta, Mark Peters, Nigel Klein, Parvez Moondi, Justin O’Grady, Juliet High, Charlotte Russell, Ann Marie Swart, David Turner, Suveer Singh

**Affiliations:** 1 Centre for Behavioural Medicine, University College London, British Medical Association House, Tavistock Square, London, WC1H 9JP UK; 2 UCLH-UCL Centre for Medicines Optimisation Research and Education, University College London Hospitals NHS Foundation Trust, London, UK; 3 Department of Psychological and Behavioural Science, London School of Economics and Political Science, London, UK; 4 Division of Critical Care, University College London Hospitals NHS Foundation Trust, London, UK; 5 Division of Infection and Immunity, University College London Faculty of Medical Sciences, London, UK; 6 University of East Anglia Norwich Medical School, Norwich, Norfolk UK; 7 Department of Medical Microbiology, University College London Hospitals NHS Foundation Trust, London, UK; 8 Department of Surgery and Cancer, Imperial College London, London, UK

**Keywords:** Rapid molecular diagnostics, Antimicrobial prescription, Intensive care

## Abstract

**Background:**

Rapid molecular diagnostic tests to investigate the microbial aetiology of pneumonias may improve treatment and antimicrobial stewardship in intensive care units (ICUs). Clinicians’ endorsement and uptake of these tests is crucial to maximise engagement; however, adoption may be impeded if users harbour unaddressed concerns or if device usage is incompatible with local practice. Accordingly, we strove to identify ICU clinicians’ beliefs about molecular diagnostic tests for pneumonias before implementation at the point-of-care.

**Methods:**

We conducted semi-structured interviews with 35 critical care doctors working in four ICUs in the United Kingdom. A clinical vignette depicting a fictitious patient with signs of pneumonia was used to explore clinicians’ beliefs about the importance of molecular diagnostics and their concerns. Data were analysed thematically.

**Results:**

Clinicians’ beliefs about molecular tests could be grouped into two categories: perceived potential of molecular diagnostics to improve antibiotic prescribing (Molecular Diagnostic Necessity) and concerns about how the test results could be implemented into practice (Molecular Diagnostic Concerns). Molecular Diagnostic Necessity stemmed from beliefs that positive results would facilitate targeted antimicrobial therapy; that negative results would signal the absence of a pathogen, and consequently that having the molecular diagnostic results would bolster clinicians’ prescribing confidence. Molecular Diagnostic Concerns included unfamiliarity with the device’s capabilities, worry that it would detect non-pathogenic bacteria, uncertainty whether it would fail to detect pathogens, and discomfort with withholding antibiotics until receiving molecular test results.

**Conclusions:**

Clinicians believed rapid molecular diagnostics for pneumonias were potentially important and were open to using them; however, they harboured concerns about the tests’ capabilities and integration into clinical practice. Implementation strategies should bolster users’ necessity beliefs while reducing their concerns; this can be accomplished by publicising the tests’ purpose and benefits, identifying and addressing clinicians’ misconceptions, establishing a trial period for first-hand familiarisation, and emphasising that, with a swift (e.g., 60–90 min) test, antibiotics can be started and refined after molecular diagnostic results become available.

**Supplementary Information:**

The online version contains supplementary material available at 10.1186/s13756-021-00961-4.

## Background

Rapid molecular diagnostic tests for pneumonia pathogens may improve antimicrobial stewardship (AMS). Results become available within 1 to 6 h, with accurate detection of multiple respiratory bacteria, viruses, and antimicrobial resistance genes directly from respiratory secretions without culture [1]. Commercially available rapid tests for pneumonia presently comprise the BioFire FilmArray Pneumonia panel (bioMérieux), the Unyvero Hospitalised Pneumonia panel (Curetis) and the FTD Respiratory Pathogens 33 (Fast Track Diagnostics) [1]. Other tests are in development.

Such tests may be particularly useful in intensive care units (ICUs), where patients with pneumonia are frequent [2, 3], there is an increase the risk of rapid deterioration and death [3, 4], and thus there is a demand for urgent antimicrobial treatment [5, 6]. Recommended practice for a suspected pneumonia include the prescription of empiric broad-spectrum antibiotics, with refinement once results of laboratory cultures become available (typically after 48–72 h) [5]. This approach is improvable because: (a) unnecessary antibiotics increase the risk of adverse consequences including direct toxicity, drug interactions, and *Clostridium difficile* infection [7], (b) empirical cover may prove ineffective for patients with drug resistant organisms [8], and (c) liberal broad-spectrum use drives antimicrobial resistance (AMR).

Molecular diagnostics can identify pathogens and their resistance genes within hours rather than days, potentially directing early tailored antimicrobial therapy [9]. Randomised-control trials (RCTs) are comparing outcomes and AMS in molecular diagnostic and conventional microbiology-guided antimicrobial treatment for pneumonias [1]. For example, INHALE (ISRCTN16483855) has implemented the evaluation of point-of-care molecular tests for pneumonias in 12 UK ICUs [10].

Clinicians’ endorsement and uptake of molecular tests is crucial to maximise engagement in the context of such a RCT and for any subsequent deployment. Yet, adoption may be impeded if users harbour unaddressed concerns or if device usage is incompatible with local practice [11]. For instance, a recent RCT found that a highly sensitive rule-out test did not improve AMS for ICU patients with suspected VAP, likely due to incongruity with local prescribing culture [12].

Clinicians’ beliefs about molecular diagnostics remain largely unknown, with the UK Department of Health and Social Care identifying a lack of understanding frontline needs as a potential delayer to the clinical adoption of molecular tests [13]. Accordingly, we explored intensivists’ beliefs of molecular diagnostics before the tests’ implementation for the INHALE RCT.

## Methods

This study is reported following Standards for Reporting Qualitative Research guidelines [14]. It employed vignette-based interviews (VBIs) using an interpretivist approach to understand clinicians’ beliefs of molecular diagnostics as a decision aid. The data are derived from the same interview transcripts as Pandolfo and colleagues [15], but were analysed separately and meet Fine and Kurdek’s criteria for publishing multiple reports from one dataset [16].

### Setting

Interviews occurred in four UK ICUs that varied in patient population and prevalence of multi-drug resistant organisms. One was a large district general hospital in Norfolk; the remainder were London-based and comprised a tertiary referral hospital, a paediatric hospital, and a private hospital with an international patient population. None had molecular diagnostic tests for pneumonia available at the time of this research.

### Inclusion criteria

All intensivists practicing at the four participating ICUs were eligible to participate. Clinicians who could spare time from clinical duties were recruited via local promotion after ward rounds.

This research received Health Research Authority approval before data collection; all participants gave written informed consent.

### Vignette-based interview methodology

Semi-structured VBIs explored clinicians’ antibiotic decision-making processes. Participants read a vignette depicting a hypothetical patient exhibiting signs of pneumonia (see Additional file 1 for vignette and interview guide). They then applied their expertise to determine whether to wait or to start antimicrobial treatment. We explored the perceived utility of molecular test results for this patient, followed by further detailed discussions of issues raised. Since participants may not have been familiar with specific devices, interviews asked generally about molecular tests for pneumonia. We posited a notional six-hour result turnaround time because this is achievable with two devices considered for the INHALE RCT. The vignette was pilot-tested with five non-participating ICU consultants and had no ‘correct’ answers because its purpose was to encourage reflection on decision-making processes.

Interviews were audio-recorded and conducted face-to-face in each site’s ICU by NB and YJ. Both have clinical pharmacy backgrounds, with qualitative research and interviewing experience.

### Analysis

Interviews were anonymised, professionally transcribed, and entered into NVivo V.12. AMP (a research psychologist), NB, and YJ verified transcription accuracy. Data were analysed by AMP and NB using thematic analysis, following Braun and Clarke’s recommendations [17].

We adopted an inductive approach whereby data were coded to capture themes that represented a pattern of responses across transcripts. Themes were then deductively mapped onto the Necessity-Concerns Framework (NCF). NCF proposes that patients’ adherence to recommended treatment plans is influenced by their beliefs about the importance of their treatment and their treatment concerns [18]. Consistent with Pandolfo and colleagues [15], we adapted the principles of NCF to explore intensivists’ beliefs regarding molecular diagnostics as a decision aid for their *patients’* treatments. ‘Molecular Diagnostic Necessity’ refers to clinicians’ perceived importance of molecular diagnostic results in practice while ‘Molecular Diagnostic Concerns’ are their beliefs about the consequences associated with test adoption [19].

## Results

Interviews occurred between August and December 2018, were between seven and 20 min in length, and enrolment continued until data saturation [20]. Total audio-recording duration was approximately 4.5 h.

Participants comprised seven early-career trainees, sixteen middle-grade trainees, and eleven consultants. Eleven participants were employed at Hospital 1, ten at Hospital 2, seven at Hospital 3, and six at Hospital 4. All intensivists practicing at Hospital 2 treated children and neonates; remaining participants treated adult patients. Interviews were individual except in one case where one early-career trainee and one middle-grade trainee were interviewed together.

We describe clinicians’ perceived importance of molecular diagnostics followed by their concerns. Tables 1 and 2 show sub-themes and supporting quotations for Molecular Diagnostic Necessity and Molecular Diagnostic Concerns themes, respectively. There were no notable differences between paediatric- and adult-treating doctors’ beliefs.

**Table 1:** Supporting quotations for Molecular Diagnostic Necessity theme

Subtheme	Supporting quotations
Positive results facilitate choosing targeted antibiotics	1. [Molecular diagnostics] would provide something more diagnostic than just a non-specific CRP or white cells that could guide specific antibiotic choice if we wanted to start them. Because, of course, in most cases, we just have to choose the most appropriate antibiotic based on what we think is the cause, which, depending on how concerned we are about the patient, it would be… the more concerned you are about them, or the more unwell they look, the broader-spectrum antibiotic you might choose. So, it could be helpful to perhaps not just start them on meropenem or Tazocin [Piperacillin/Tazobactam]. *–P15, middle-grade trainee, Hospital 3*
	2. […] if you had something [i.e., molecular diagnostics] that could give you quicker results you could give someone more appropriate antibiotics from the start. *–P20, early-career trainee, Hospital 4*
	3. [Molecular diagnostics] would mean your rationalisation of your antibiotic regime would be much faster. You’d be able to switch to one with the correct sensitivities within six hours rather than 12 to 24, which would be much better. –*P21, middle-grade trainee, Hospital 3*
Positive results lower threshold for starting antibiotics	4. […] if I had proof that there was a bacteria [*sic*] in someone who was spiking a temperature, then I think I would be more likely to start antibiotics. […] In the same way if I had a positive urine dip or a consolidation on a chest X-ray. *–P44, middle-grade trainee, Hospital 1*
	5. […] even if there is a positive result in the test I would still think about starting it [antibiotics]. I wouldn’t start it automatically. –*P8, consultant, Hospital 4*
	6. [Molecular diagnostics] might change what [antibiotic] I give but it wouldn’t decide whether I give anything. –*P29, consultant, Hospital 4*
	7. For starting antibiotics, it would probably not be helpful to know the microbiology [i.e., molecular diagnostic results] as such. Because for starting antibiotics, or for a decision of which antibiotics, you need to have a context. Once you know that the patient is deteriorating in some way, and the context is triggering the confirmation that this is not inflammation, it is actually infection, obviously, to know what is going on in the body, with a name with a species, can make a huge difference, definitely. –*P1, consultant, Hospital 3*
Negative results indicate absence of respiratory infection	8. […] if that [molecular test result] was negative, I wouldn’t give her [vignette patient] any antibiotics. –*P35, senior trainee, Hospital 2*
	9. If that [molecular test] comes up negative and I’ve got a patient who, yes, at the moment doesn’t look particularly infected, probably just still sit, watch and wait and see. […] But, a clear negative in a patient who’s otherwise now looking to be frankly septic, more septic than this does, then it might change the spectrum antibiotics I want to consider. So, say that [central] line was relatively new, so maybe I’m more suspicious of the line than I would have been otherwise. […] where it’s clearly negative, then it would change my thinking around other likely sources of infection. –*P48, consultant, Hospital 1*
Molecular diagnostic results increase confidence in prescribing decisions	10. [Having molecular test results] would mean that you can be confident that you’ve identified a pathogen that is sensitive, and that you could use a more narrow-spectrum antibiotic to focus that pathogen that you think is responsible for the sepsis, rather than having a bit of a panic using a broad-spectrum antibiotic. […] [Molecular results will] help a lot in terms of keeping the more powerful antibiotics for the situations in which you really need them. And that, in turn, will help to reduce the antibiotic resistance in the future. –*P53, middle-grade trainee, Hospital 3*
	11. […] [If the molecular test] comes back positive, I come out of this state of diagnostic uncertainty, I feel much happier now. Because while I’m in the waiting zone I’m in a state of angst, have I made the right decision, have I made the wrong decision? […] And so great, if I’ve made a diagnosis more quickly, it’s lovely that we remove the diagnostic angst. And I can be happy that I’m now making a good decision for the patient and by proxy a good decision for the population. I don’t have to feel bad about needlessly increasing antibiotic resistance. –*P43, consultant, Hospital 1*

*CRP* C-reactive protein, *PCR* polymerase chain reaction

**Table 2:** Supporting quotations for Molecular Diagnostic Concerns theme

Subtheme	Supporting quotations
Unfamiliarity with molecular diagnostic test capabilities	12. I need to know about the device, I think. What can it detect, in what populations has it been used, how confident can I be? We all know that I’ve got a fairly poor set of clinical tools for defining respiratory infection at the moment. But it’s before I’m going to start interpreting another one I need to know a bit more about that. –*P48, consultant, Hospital 1*
	13. I’m not familiar with this machine; I’ve survived 30 years without having one. […] because I just don’t have a feel for the machine, I’d have to try it out for a bit and see what the results are. We have to try and use evidence based, don’t we? I’d be reassured if I knew that either it worked really, really well or, on the other hand, that it didn’t. –*P36, consultant, Hospital 2*
	14. Products come in and tests come in and it doesn’t necessarily change what we’re actually doing on a day to day until we’ve seen it work a few times. –*P58, middle-grade trainee, Hospital 1*
Molecular diagnostics detecting non-pathogenic bacteria may lead to over-treatment	15. […] how convinced would I be that it’s [molecular test] picking up a pathogen rather than just an incidental coloniser. I’m not sure it would change what I’d do. –*P6, consultant, Hospital 3*
	16. […] there’s no test which is 100% specific, and there’s no test which is 100% sensitive. So yes, I’m thinking whether this [test] would lead to over-prescribing of antibiotics that might lead to an increase in drug resistance […] That’s why it’s very important to know how sensitive and specific the PCR is. There are a lot of commensals in our respiratory tract. *–P31, middle-grade trainee, Hospital 2*
	17. […] whether that [positive result] is a colonisation rather than infection it would still be the same decision-making processes. So new temperature, a change in the inflammatory markers, a change in the secretion burden. –*P45, consultant, Hospital 1*
Molecular diagnostics failing to detect pathogens may lead to under-treatment	18. […] if I’ve grown^a^ nothing I’m not sure whether that would be depending on the sensitivities or whether that’d be reassuring enough to not cover the chest and just cover the abdomen. –*P58, middle-grade trainee, Hospital 1*
	19. If she [vignette patient] didn’t bring up anything [i.e., negative result], but we still suspected a chest infection, or the X-ray showed something, then we would start [antibiotics]. –*P2, early-career trainee, Hospital 4*
Concern of patient deterioration while awaiting molecular diagnostic results	20. […] six hours doesn’t seem that long. But that could have a detrimental effect because six hours could be too long for this patient. –*P24, early-career trainee, Hospital 1*
	21. If I thought it [molecular results] was something that was very convincing and she [vignette patient] was quite stable and I could wait two hours, then yes, potentially. But it doesn’t sound like you’re portraying a stable case; you’re portraying someone that has come from a local unit looking like a bronchiolitis, getting worse, needing intubating. –*P54, middle-grade trainee, Hospital 2*
	22. I don't think you could justify waiting six hours to treat someone if they’ve got overt signs of sepsis. –*P21, middle-grade trainee, Hospital 3*
	23. I wouldn’t start [antibiotics], I would wait a few hours because if I know in a couple of hours I’d have a result and she’s [vignette patient] not in shock and not very bad I would wait to see if something comes up positive. –*P23, middle-grade trainee, Hospital 2*
	24. Because if you knew what the answer would be within six hours, and given that she’s [vignette patient] not systemically unwell at the moment, I would be more comfortable holding off [antibiotics]. –*P47, early-career trainee, Hospital 4*

*PCR* polymerase chain reaction

^a^This is a misapprehension; the device does not ‘grow’

### Molecular Diagnostic Necessity: molecular diagnostic results improve antibiotic prescription practices

#### Positive results facilitate choosing targeted antibiotics

Positive molecular diagnostic results (i.e., detection of bacterial pathogen by the test) were generally believed to facilitate antibiotic choice by rapidly identifying the organism(s) and predicting their antibiotic susceptibilities (Quotes 1–3). Currently, this information would be available approximately 72 h after the initial prescribing decision, leading to lengthy courses of potentially sub-optimal therapy. Many participants felt that molecular test results would encourage either starting appropriate antibiotics at the outset or swiftly de-escalating empirical therapy to narrow-spectrum antibiotics.

#### Positive results lower threshold for starting antibiotics

In addition to improving antibiotic choice, ten clinicians asserted that positive results would *lower their threshold* for starting antibiotics. For example, one trainee likened molecular diagnostics to other infection indicators (e.g., white cell count in blood) which give “*proof*” of an infection but should be combined with the clinical context before making an antimicrobial decision (Quote 4). This decision was frequently described as reflecting a combination of clinical factors, including prospectively, the molecular test result. None of our participants said that they would start antibiotics solely based on positive results (Quote 5).

Some doctors stated that molecular diagnostics would not influence their threshold to start antimicrobial therapy (Quote 6). These clinicians believed that they would exclusively use this test to choose *appropriate antibiotics* because their decision to start antibiotics would only be based on the clinical context (Quote 7).

#### Negative results indicate absence of respiratory infection

Negative results (i.e., no detection of bacteria or resistance genes) would generally be interpreted to indicate that a respiratory infection was unlikely. Some clinicians believed that a negative result in a clinically stable patient would encourage withholding or stopping antibiotics (Quotes 8–9), whereas a negative result in a deteriorating patient would be interpreted to indicate a non-respiratory source of infection (Quote 9).

#### Molecular diagnostic results increase confidence in prescribing decisions

When currently making antibiotic decisions, some participants reported experiencing negative emotions like “*angst*” and “*panic*” (Quotes 10–11). They described uncertainty regarding whether their prescribing was appropriate and worried that it contributed to AMR (Quotes 10–11). This reflects the present difficulty of verifying the appropriateness of antibiotic decisions until laboratory culture results become available.

Such participants believed that the information provided by the molecular test would make them “*happier*” and more “*confident*” in their prescribing (Quotes 10–11). They believed that their prescribing decisions would be beneficial to their patient and society and felt that access to these tests would encourage AMS practices.

### Molecular Diagnostic Concerns: integrating molecular diagnostics into practice

#### Unfamiliarity with molecular diagnostic test capabilities

Many clinicians stated that they needed to familiarise themselves with the molecular test before using its results in practice. First, they wanted more information about the test, including its sensitivity, specificity, and its place in the diagnostic process (Quote 12). Several raised misconceptions about the test’s capabilities (e.g., it would prove the absence of all possible resistance genes and mutations; Quote 10). Second, they wanted first-hand experience to verify the test’s suitability (Quote 13). These participants felt a sense of *caveat emptor*—as future users of the test’s results, these clinicians believed that familiarisation was essential to understand the test’s capabilities and to make an informed decision about its usefulness in practice (Quotes 13–14). These participants would not adopt the test if, after familiarisation, they concluded that it did not meet their standards or would negatively impact patient care.

#### Molecular diagnostics detecting non-pathogenic bacteria may lead to over-treatment

Concerns were common that the molecular test would detect organisms present, but of no consequence to the patient (Quote 15). Many clinicians worried that the test would detect non-pathogenic bacteria, and that, paradoxically, this might encourage unnecessary antibiotic prescriptions (Quote 16). To mitigate against antibiotic over-treatment based on detection of colonisation rather than infection, some participants stressed that they would not automatically prescribe based on the test result. They would combine the results with the clinical context to determine the likelihood that the bacteria detected were causing current disease before making a decision (Quote 17).

#### Molecular diagnostics failing to detect pathogens may lead to under-treatment

Some clinicians reported that negative results would not be reassuring enough to withhold or stop antibiotics. These doctors were uncertain whether the test was able to detect all possible respiratory pathogens (Quote 18). They consequently believed that negative results should be overridden by other evidence of respiratory infection (e.g., chest X-ray consolidation), which would lead them to prescribe or continue antibiotics (Quote 19).

#### Concern of patient deterioration while awaiting molecular diagnostic results

Many early-career and middle-grade trainees assumed that they would need to withhold antibiotics until molecular test results become available; no consultants took this view. Consequently, some junior doctors expressed discomfort with the prospect of withholding antibiotics for up to 6 h. They worried that both the vignette and their actual patients would significantly deteriorate within this period (Quotes 20–21), believing that withholding antibiotics would be indefensible if the patient was exhibiting signs of sepsis (Quote 22).

Other trainees felt more comfortable with withholding antibiotics until receiving molecular test results. These participants believed that the patient was stable enough to ‘wait and watch’ and valued using the results to aid their ultimate prescription decision (Quotes 23–24).

## Discussion

This is the first study exploring UK intensivists’ salient beliefs influencing molecular diagnostic test uptake. Consistent with the NCF, facilitators related to clinicians’ beliefs that molecular tests would improve AMS whereas barriers related to their concerns about integrating these tests into clinical practice [19].

Many participants were open to using the molecular test because they believed that its results would improve antibiotic prescription practices. They saw positive test results, with early specific identification of a pathogen, as facilitating targeted antimicrobial therapy. Similarly, they viewed negative results to signal the likely absence of respiratory infection and thought that these results would provide reassurance that withholding an antibiotic prescription was appropriate. Our findings are consistent with arguments that molecular diagnostics would aid the optimisation of antimicrobial therapy [1, 9], and that it could balance the competing priorities of individual patients with AMR [21, 22].

Despite clinicians recognising the potential of molecular tests, concerns about tests’ integration into clinical practice may impede uptake. Consistent with previous assertions [1, 21], participants worried that a molecular test’s high sensitivity would lead to reporting of colonising bacteria, which, paradoxically, could prompt unnecessary antimicrobial therapy. They also raised concerns about unfamiliarity with the test, misapprehensions about its capabilities, uncertainty whether it would fail to detect pathogens, and worry about withholding antibiotics until receiving test results. Clinicians may also hold these beliefs regarding the ability of conventional microbiology to find organisms, especially when antibiotics have been deployed [23].

Individual and environmental factors may also influence prescribers’ acceptance of molecular diagnostics. Individual clinicians’ trust in molecular diagnostics may affect its usage; for example, prescribers with low levels of trust in the machine likely would harbour concerns while having low beliefs about the device’s utility. Similarly, the prescribing environment may affect machine uptake. For instance, unit-specific norms may encourage continuing empiric antibiotics for a minimum course; as such, receiving rapid test results may not impact antibiotic de-escalation or stopping. Thought should also be given to machine placement, whether at the point-of-care or in the laboratory.

Our findings show the importance of understanding clinicians’ beliefs about molecular diagnostic tests to ensure that they understand their rationale, and to sufficiently address their concerns. To encourage molecular diagnostic uptake in ICU—whether for RCTs or for permanent use—implementation strategies should bolster users’ necessity beliefs while reducing concerns; this may be achieved with relatively simple solutions (summarised in Table 3).

**Table 3:** Recommendations to facilitate molecular test uptake

Concern raised during interview	Recommendation
Unfamiliarity with molecular diagnostics	Publicise the technology’s purpose and benefits, particularly its rapidity, accuracy, and ability to facilitate targeted antimicrobial therapy
	Identify and address any misconceptions held by clinicians
	Establish a trial period for clinician first-hand familiarisation
Withholding antibiotics until receiving molecular diagnostic results	Emphasise that empiric antibiotics can be started and refined after molecular diagnostic results become available

This study has limitations. Firstly, our interviews were conducted before the molecular diagnostic test was deployed for the INHALE trial. Different beliefs may emerge after implementation and should be explored. Secondly, we interviewed ICU intensivists as the INHALE RCT runs with the molecular test located in ICUs; however some clinicians have recommended collaborating with microbiologists during test implementation [21, 24]. Future research will investigate ICU microbiologists’ beliefs of molecular diagnostics to understand their barriers and facilitators to adoption. The INHALE team has also collaborated with unit microbiologists to create site-specific prescribing algorithms that aid translation of machine output to prescribing advice. Thirdly, clinicians’ beliefs may differ for non-respiratory molecular tests and should be examined in future research. Lastly, while we have endeavoured to sample from diverse hospitals, we recognise that clinicians’ beliefs may vary elsewhere in the UK and in other countries.

## Conclusions

This study is the first to characterise prescribers’ beliefs about molecular diagnostics as facilitators and barriers to applying it to antibiotic prescribing for patients with suspected pneumonia. Our findings demonstrate the importance of prescribers’ beliefs as determinants of molecular diagnostic uptake. If this technology proves successful in improving antibiotic prescribing and stewardship, interventions should understand and address these beliefs to ensure the optimal application of molecular diagnostics in clinical practice.

## Supplementary Information

**Additional file 1.** Interview guide and vignettes.

## Data Availability

No data are available.

## Abbreviations

AMR: Antimicrobial resistance
AMS: Antimicrobial stewardship
ICU: Intensive care unit
NCF: Necessity Concerns Framework
RCT: Randomised-control trial
UK: United Kingdom
VBI: Vignette-based interview
