# Supplementary material for: Intensivists’ beliefs about rapid multiplex molecular diagnostic testing and its potential role in improving prescribing decisions and antimicrobial stewardship: a qualitative study
Source: Antimicrob Resist Infect Control. 2021 Jun 29;10:95. doi: 10.1186/s13756-021-00961-4 (PMC8243627; doi:10.1186/s13756-021-00961-4)
Supplement: Supplementary file 1 — Additional file 1. Interview guide and vignettes. [file 13756_2021_961_MOESM1_ESM.docx]

Supplementary Table 1: Interview guide and vignettes

| **A starting vignette (Adult)**  Frieda is 55yo [years old] and was admitted to your ICU [intensive care unit] 10 days ago with acute, severe pancreatitis. She has suffered in the past from gall stones but apart from intermittent pain they caused her no other problems. However, 12 days ago she presented to the ED [emergency department] with very severe abdominal pain and a diagnosis of (necrotising) pancreatitis was confirmed by a CT [computerized tomography] scan and a markedly raised amylase. Following admission she deteriorated on the ward with increasing abdominal distension, progressive cardiovascular and respiratory deterioration. She required intubation and ventilation and, for 48 hours required noradrenaline. She was started on a course of meropenem and TPN [total parenteral nutrition] (intravenous food) via a central line at the time of intubation. Otherwise management was conservative.  10 days later and things are improving, she is no longer on vaso-actives, her abdomen is less distended and NG [nasogastric] aspirates are decreasing, though the surgeons still feel TPN is warranted. She remains intubated but is slowly weaning from the ventilator (FiO2 [fraction of inspired oxygen] 0.45, PSV [pressure support ventilation] 15/7) she is slowly waking up as the sedation is reduced. The meropenem was stopped 3 days ago.  You are covering the ICU tonight and the nurse at the bedside reports she has a new temperature of 38.1C. The chart shows no other temperatures over the last 4 days. There was nothing unusual about her morning bloods (CRP [C-reactive protein]/WCC [white cell count], raised but stable etc.). She is also a bit more tachycardic, normally ~90bpm [beats per minute], now ~100bpm but blood pressure is maintained, urine output seems good and no change to her respiratory parameters or blood gas.  Her CVC [central venous catheter] looks clean, the abdomen is distended and appears tender on palpation (though no more than last night), and she has decreased air entry at both bases with occasional coarse crackles. | **Interview guide**   1. What is going through your mind here?^[[1]](#footnote-1)^ 2. Would you start antibiotics? 3. How comfortable are you with your decision? 4. If you are not 100% sure you are doing the right thing what information would you like (either existing tests or one from the future)? 5. If you had a device able to detect respiratory pathogens in 6 hours, would that change your approach (or levels of comfort)? 6. Does antimicrobial stewardship factor into your prescribing decisions? In what ways? |
| --- | --- |
| **A starting vignette (Paediatric)**  Amber is an 11 month old admitted to your ICU with respiratory distress. She had been unwell for 48 hours with a mild temperature, cough and fast breathing. She was seen in A and E [Accident and Emergency] at her local hospital and referred to [Hospital name] for concerns about breathing. Her respiratory distress worsened and her oxygen Sats [saturation] started to decline following arrival on PICU [paediatric intensive care unit]. She had bilateral crackles and wheezes. An NPA [nasopharyngeal airway] from the referring hospital revealed RSV [respiratory syncytial virus]. The Intensivists felt she required intubation and ventilation. A CXR [chest X-ray] showed some patchy shadowing. She has a fever of 38.5C. Her CRP is 22. |  |

1. Participants’ responses to questions 1, 2, 3, and 6 have been analysed and reported separately. [↑](#footnote-ref-1)
